# Host-Malaria Parasite Interactions and Impacts on Mutual Evolution

**DOI:** 10.3389/fcimb.2020.587933

**Published:** 2020-10-27

**Authors:** Xin-zhuan Su, Cui Zhang, Deirdre A. Joy

**Affiliations:** ^1^Laboratory of Malaria and Vector Research, National Institute of Allergy and Infectious Diseases, National Institutes of Health, Bethesda, MD, United States; ^2^Parasitology and International Programs Branch, National Institute of Allergy and Infectious Diseases, National Institutes of Health, Bethesda, MD, United States

**Keywords:** *Plasmodium*, genome diversity, population, vaccine, immunity, selection

## Abstract

Malaria is the most deadly parasitic disease, affecting hundreds of millions of people worldwide. Malaria parasites have been associated with their hosts for millions of years. During the long history of host-parasite co-evolution, both parasites and hosts have applied pressure on each other through complex host-parasite molecular interactions. Whereas the hosts activate various immune mechanisms to remove parasites during an infection, the parasites attempt to evade host immunity by diversifying their genome and switching expression of targets of the host immune system. Human intervention to control the disease such as antimalarial drugs and vaccination can greatly alter parasite population dynamics and evolution, particularly the massive applications of antimalarial drugs in recent human history. Vaccination is likely the best method to prevent the disease; however, a partially protective vaccine may have unwanted consequences that require further investigation. Studies of host-parasite interactions and co-evolution will provide important information for designing safe and effective vaccines and for preventing drug resistance. In this essay, we will discuss some interesting molecules involved in host-parasite interactions, including important parasite antigens. We also discuss subjects relevant to drug and vaccine development and some approaches for studying host-parasite interactions.

## Introduction

### Malaria Parasites, Life Cycle, and Genome

Malaria is a parasitic disease caused by *Plasmodium* species (spp.), unicellular protozoan organisms in the phylum of *Apicomplexa*. The species that infect humans include *Plasmodium falciparum, P. vivax, P. malariae, P. knowlesi*, and *P. ovale*, with *P. ovale* recently recognized as two sub-species called *Plasmodium ovale curtisi* (classic type) and *Plasmodium ovale wallikeri* (Sutherland et al., 2010). Whereas, *P. vivax* is the most widespread species, *P. falciparum* is the most deadly to humans. There are several other species of malaria parasites that infect non-human primates including multiple species that infect African apes, and others that infect Asian and New World monkeys (Escalante et al., 2005; Liu et al., 2010; Boundenga et al., 2015; Martinelli and Culleton, 2018; Galinski, 2019). There are also malaria parasites of rodents such as *P. berghei, P. chabaudi*, and *P. yoelii*, often used as animal models of malaria, as well as parasites that infect birds (*P. gallinaceum* and *P. relictum*) (Duval and Ariey, 2012; Ramiro et al., 2012; Fecchio et al., 2020), lizards, bats, and ungulates (Schall, 1982; Schaer et al., 2013; Templeton et al., 2016; Galen et al., 2018). Human malaria cases are mostly reported in tropical and subtropical regions, particularly in Africa, south and southeast Asia, and south and central America. In 2018, an estimated 228 million cases of malaria occurred worldwide, with ~213 million (or 93%) in Africa (WHO, 2019).

**Figure 1:**
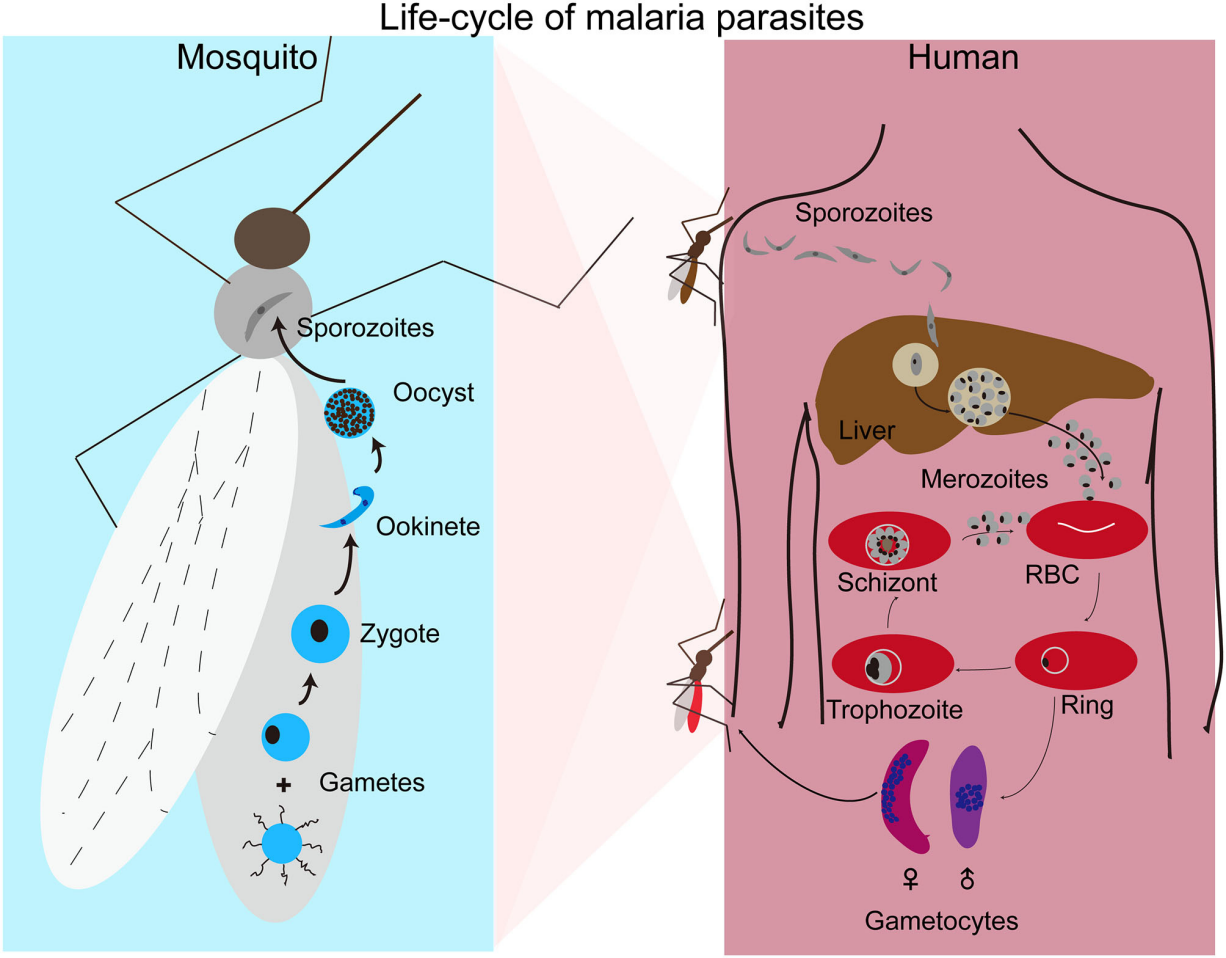
Life cycle of malaria parasites. Malaria parasite infection starts with a mosquito bite injecting sporozoites into human body where the parasites travel to liver and invade hepatocytes. After replication in liver cells, thousands of mature merozoites are released into blood stream and invade red blood cells (RBCs). The parasites then replicate in RBCs that subsequently rupture, releasing more merozoites to invade new RBCs for another cycle. Malaria symptoms correlate with the rupture of infected RBCs. A small number of merozoites develop into male and female gametocytes after invading new RBCs. Gametocytes differentiate into male and female gametes in the mosquito midgut when another mosquito takes a blood meal. Male and female gametes fertilize to produce zygotes that develop into motile ookinetes and oocysts after penetrating the mosquito midgut. A large number of sporozoites from each oocysts moves to salivary glands and are injected into a new human host when the mosquito bites again.

Human malaria infections start with a bite from an infected female *Anopheles* mosquito, injecting sporozoites into the skin of a host (Figure 1). The sporozoites move to the liver through blood vessels and then traverse liver sinusoidal endothelial cells or Kupffer cells to infect hepatocytes (Baer et al., 2007; Tavares et al., 2013). Inside a hepatocyte, a parasite divides into thousands of merozoites through a process called schizogony. Mature merozoites enter the bloodstream after rupture of the infected hepatocyte and invade erythrocytes, starting a new cycles of schizogony within red blood cells (RBCs) which includes asexual replication of their haploid genome. Within RBCs parasites develop through ring, trophozoite, and schizont stages. The resulting mature schizont is segmented in appearance and contains 16–32 daughter merozoites. The infected RBC (iRBC) ruptures, releasing the daughter sporozoites to invade new RBCs. This cycle of erythrocytic development takes approximately 24 h for *P. knowlesi*, 48 h for *P. falciparum, P. vivax*, and *P. ovale*, and 72 h for *P. malariae*. In addition to merozoites, ruptured iRBCs also release various by-products of parasite metabolism such as hemozoin formed when the parasite digests hemoglobin. The release of parasite materials also triggers host responses leading to various clinical symptoms, including fever, chills, headache, dizziness, back pain, and myalgia (Ashley et al., 2018). Some patients may progress to severe malaria with coma (cerebral malaria), pulmonary edema, acute renal failure, jaundice, severe anemia, acidosis, hypoglycemia, and death (Trampuz et al., 2003; Sypniewska et al., 2017; Luzolo and Ngoyi, 2019). In response to changes in the host environment such as variation in metabolites and immune factors (Josling and Llinas, 2015; Brancucci et al., 2017), some of the parasites differentiate into sexual stages termed male and female gametocytes. When a mosquito takes a bloodmeal from an infected host, gametocytes will undergo sexual development in the midgut of the mosquito. Within minutes of entering the mosquito midgut, male and female gametocytes differentiate into male and female gametes that fertilize to form zygotes and then into motile ookinetes. Ookinetes penetrate through the mosquito midgut wall to develop into oocysts containing thousands of sporozoites. Mature sporozoites migrate to salivary glands and are injected into a new vertebrate host when the mosquito bites again, completing the life cycle.

The genomes of many malaria parasites infecting humans (*P. falciparum, P. vivax, P. ovale, P. malariae, P. knowlesi*), non-human primates (*P. reichenowi, P. praefalciparum, P. blacklocki, P. adleri, P. billcollinsi, P. gaboni, P. cynomolgi, P. coatneyi, P. inui, and P. fragile*), rodents (*P. berghei, P. yoelii, P. chabaudi*, and *P. vinkei*) and birds (*P. relictum* and *P. gallinaceum*) have been sequenced and analyzed (Shutler et al., 2005; Pain et al., 2008; Otto et al., 2014a,b, 2018; Lauron et al., 2015; Ansari et al., 2016; Auburn et al., 2016; Sundararaman et al., 2016; Pasini et al., 2017; Rutledge et al., 2017; Bohme et al., 2018; Su et al., 2019). Malaria parasites have relatively small haploid genomes, ranging from 20 to 35 megabases (Mb) that contain 14 chromosomes, a circular plastid genome of ~35 kb, and multiple copies of a 6-kb mitochondrial DNA (Gardner et al., 2002; Otto et al., 2014a,b; Auburn et al., 2016; Pasini et al., 2017; Bohme et al., 2018). The homologous genes in different species of malaria parasites are often found in syntenic blocks arranged in different order on the chromosomes (Carlton et al., 2005; Kooij et al., 2005). For more information on *Plasmodium* genome and genomics associated with parasite development, diseases, diagnosis, epidemiology, and vaccine development please consult (Kirchner et al., 2016; Bourgard et al., 2018; Escalante and Pacheco, 2019; Galinski, 2019; Garrido-Cardenas et al., 2019; Su et al., 2019; Videvall, 2019).

## Evolution and Adaptation of Malaria Parasites in Great Apes

### Origin of *P. falciparum*

Our understanding of the host-parasite interactions involved in the emergence of *P. falciparum* in humans has been greatly enabled by the discovery of multiple closely related malaria species in African apes beginning in the early 2000s. Three malaria species morphologically similar to *P. falciparum* were described in African apes during the last century (Reichenow, 1920; Blacklock and Adler, 1922; Adler, 1923), and of these only one, *P. reichenowi*, had been sequenced (Neafsey et al., 2005). This picture began to change with the discovery of a new malaria species identified in chimpanzees kept as pets in a village in Gabon (Ollomo et al., 2009). Based on the complete mitochondrial genome this parasite was found to fall within the *Laverania* subgenus that includes *P. reichenowi* and *P. falciparum*. A number of other studies quickly followed, leading to the discovery of additional novel malaria species, and a rethinking of the origin of *P. falciparum* in humans. DNA sequences of the cytochrome oxidase b (*cyt b*) gene in samples from a combination of both wild-living and captive chimpanzees and gorillas showed an unexpectedly diverse collection of *Plasmodium* lineages circulating in African apes, suggesting that *P. falciparum* likely originated from *P. reichenowi* via a single transfer event from chimpanzees to humans (Prugnolle et al., 2010). Sequencing of the *Plasmodium* mitochondrial genome and two nuclear genes, dihydrofolate reductase-thymidylate synthase (*dhfr-ts*) and the gene encoding the merozoite surface protein 2 (*msp2*), from blood samples of captive chimpanzees and bonobos in Uganda and the Democratic Republic of Congo identified two new *Plasmodium* species, and led the authors to conclude that *P. falciparum* arose in bonobos (*Pan paniscus*) (Krief et al., 2010). Yet another study of a small number of chimpanzees and gorillas from Cameroon previously kept as pets targeted two mitochondrial genes (*cytb* and *cox1*), one plastid gene (*tufA*), and one nuclear gene (*ldh*), and purportedly found *P. falciparum* in both chimpanzee and gorilla blood samples (Duval et al., 2010).

**Figure 2:**
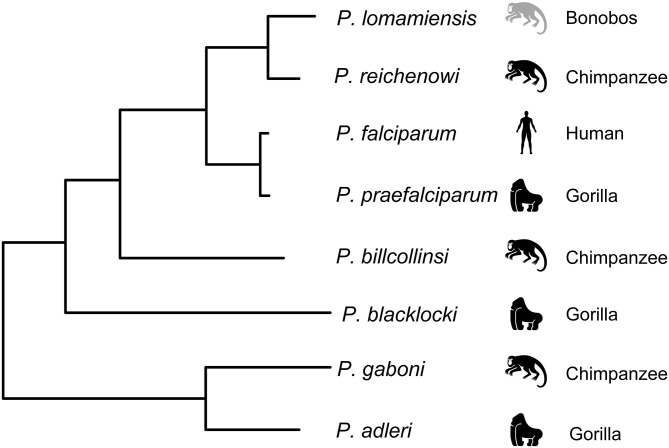
Relationship among the *Laverania* species, adapted from Figure 1A in Plenderleith et al. (2019). Icons indicate the parasites usual host species: gorillas (*Gorilla gorilla*), chimpanzees (*Pan troglodytes*), bonobos (*Pan paniscus*), and humans. The tree was derived by maximum likelihood analysis of a 3.4 kb region of the mitochondrial genome (see Supplementary Figure 1A in Liu et al., 2010).

The contradictory findings on the origin of *P. falciparum* could be explained in part by sampling limitations, including small sample sizes and a general reliance on captive animals which introduces the possibility of contemporary infection by humans. These concerns were addressed in a large-scale study of non-invasive samples from wild-living chimpanzees, gorillas, and bonobos throughout central Africa (Liu et al., 2010). Mitochondrial, apicoplast, and nuclear genes from over 1,000 samples were sequenced, and most infected samples were found to contain multiple genetically diverse parasites, highlighting another potential problem with the previous studies. In samples with multiple genetically diverse parasites, conventional PCR methods risk generating recombinant artifacts that could mislead the data. To guard against this, samples were subjected to limiting dilution such that each positive PCR reaction contained no more than a single parasite. Using this approach, the number of host-specific parasite species within the *Laverania* subgenus was increased to seven: *P. gaboni, P. billcollinsi*, and *P reichenowi* in chimpanzees; *P. praefalciparum, P. blackloci*, and *P. adleri* in gorillas; and *P. falciparum* in humans (Liu et al., 2010). One additional parasite species, *Plasmodium lomamiensis*, was later identified in bonobos (Liu et al., 2017). *P. praefalciparum* in western gorillas was found to be genetically nearly identical to *P. falciparum*, which formed a monophyletic clade (Figure 2), strongly suggesting that the *P. falciparum* derived from a host switch from gorillas to humans. Genome sequences from *P. reichenowi* and two *P. gaboni* parasites would later allow for an estimation of the genetic diversity of these two species (Sundararaman et al., 2016). Both *P. reichenowi* and *P. gaboni* were estimated to be at least 10X more diverse than *P. falciparum*, strongly suggesting a relatively recent origin for *P. falciparum*. This was confirmed when additional *Laverania* genomes were used to estimate the divergence of *P. falciparum* and *P. praefalciparum* to be 40,000–60,000 years ago (Otto et al., 2018). Additionally, the successful jump to humans by *P. falciparum* appears to have involved multiple parasites over time rather than a single event (Otto et al., 2018). The relationships of *Laverania* species based on maximum likelihood analysis of a 3.4 kb region of the mitochondrial genome are presented in Figure 2.

### Molecular Basis of Host Trophism and Adaptation to Humans

The *Laverania* exhibit strong host trophism, with each parasite species infecting a single host species, although rare host switching events have occurred. Host trophism is not seen to the same extent in malaria parasites that infect Asian primates (*P. cynomolgi* and *P. knowlesi*). Barriers restricting parasite species to a single host species could exist at any of the host-parasite-vector interfaces, although considerable attention has focused on host-parasite interactions during the blood stage, specifically erythrocyte invasion (Martin et al., 2005; Rich et al., 2009; Rayner et al., 2011; Wanaguru et al., 2013; Galaway et al., 2019; Plenderleith et al., 2019; Proto et al., 2019).

The production of a greatly improved *P. reichenowi* reference genome, as well as a partial genome of *P. gaboni*, allowed for the use of comparative genomics to look for genetic loci involved in host restriction. The genomes of *P. reichenowi, P. gaboni*, and *P. falciparum* showed good conservation and synteny, but with striking differences in some erythrocyte invasion loci (Otto et al., 2014b). A large-scale genomics study of *Laverania* including multiple genomes from all known species found evidence for three interspecific gene transfers (Otto et al., 2018), one which would later be challenged by a reanalysis of the sequence reads (Plenderleith et al., 2019). Several genes involved in either invasion or pathogenesis were fixed in parasites infecting the different primate hosts (Otto et al., 2018). Both a gene transfer event (Sundararaman et al., 2016) and an ancient introgression involving some erythrocyte invasion and surface protein genes (Otto et al., 2018) may have laid the groundwork for *P. falciparum's* host switch to humans. There is strong evidence of a horizontal transfer of an 8 kb region of chromosome 4 from *P. adleri* to the ancestor of *P. falciparum*, although disagreements exist as to whether the 8 kb fragment containing *Rh5* resulted from introgression or a horizontal transfer event (Sundararaman et al., 2016; Galaway et al., 2019). This region contained two erythrocyte invasion genes encoding the reticulocyte-binding-like homologous protein 5 (RH5) and the cysteine-rich protective antigen (CyRPA). RH5 differs from the other members of the RH family in that it is a secreted rather than a membrane-tethered protein and cannot be genetically deleted in any *P. falciparum*. A combination of ancestral sequence reconstruction and quantitative protein interaction assays was used to investigate the role of RH5 in the emergence of *P. falciparum* in humans (Galaway et al., 2019). Probabilistic-based approaches to deduce the likely sequence of the *rh5* gene in the common ancestor of *P. falciparum* and *P. praefalicarum* converged on a single sequence, which the authors termed the ancestral introgressed *Rh5* gene (*IntRh5*). *P. falciparum* RH5 and its orthologs were expressed as soluble recombinant proteins, and the binding affinities of RH5 orthologs for human, gorilla, and chimpanzee basigins were quantified using surface plasmon resonance (SPR) and confirmed with cell-based binding assays. Surprisingly, IntRH5 protein was found to bind to human and gorilla basigin with similar affinities. This promiscuous receptor-binding phenotype was also observed in all parasites belonging to clade A (*P. adleri* and *P. gaboni*) but not in parasites belonging to clade B (*P*. *billcollinsi, P. reichenowi, P. preafalciparum*). As *P. falciparum* is restricted to humans with no African apes having been found to be naturally infected with this parasite, the question remains as to how this restriction occurred if the ancestral character state was permissive. Others have shown that recombinant PfRH5 does not interact with gorilla basigin, and only weakly interacted with chimpanzee basigin (Wanaguru et al., 2013). Galaway et al. (2019) identified six mutations separating *IntRh5* and *PfRh5*. A single mutation at residue 200 resulted in the complete loss of PfRH5 binding to gorilla basigin, suggesting that the host switch from gorillas to humans involved at least two steps, one in which erythrocytes from both hosts could be infected, followed by restriction in humans (Otto et al., 2018).

The erythrocyte binding-like (EBL) gene family also plays a critical role in erythrocyte invasion through the recognition of glycophorins, the predominant sialoglycoprotien on the erythrocyte surface. Different EBLs recognize different gylcophorins; for example, *Pf* EBA175 recognizes glycophorin A, and invasion of human erythrocytes by *P. falciparum* is heavily reliant on this EBL. The potential for sialic acid recognition to play a role in the exclusion of *P. falciparum* from chimpanzees and *P. reichenowi* from humans was recognized and investigated prior to the discovery of multiple *Laverania* species in African apes (Martin et al., 2005). The *P. falciparum* EBL165 gene (*PfEbl165*) is a pseudogene due to a frame-shift mutation in all known *P. falciparum* isolates studied so far (Proto et al., 2019). In contrast, all *Ebl165* homologs in ape *Laverania* species encode an intact protein (Otto et al., 2014b, 2018; Sundararaman et al., 2016). Due to a mutation in the gene encoding cytidine monophospho-N-acetylneuraminic acid hydroxylase (CMAH), human erythrocytes produce only Neu5Ac sialic acids whereas ape erythrocytes produce both Neu5Ac and Neu5Gc. EBA165 binding is dependent on the specific sialic acid repertoire displayed, and the absence of ape-specific Neu5Gc sialic acid on human RBCs is sufficient to prevent PfEBL165 from binding to them (Proto et al., 2019). CRISPR-Cas9 mediated correction of the frameshift mutation in *PfEbl165* led to the epigenetic silencing of a small number of genes in two regions of chromosome 4 and 11 that included *PfEbl165* itself and *PfRh4* (another invasion gene) (Proto et al., 2019). It appears as though a functional PfEBL165 is not compatible with robust growth of *P. falciparum* in humans, suggesting that the inactivation of this gene may have contributed to the parasites' establishment in this host. As an increasing number of candidate loci are studied, it is becoming clear that a single molecular event is not sufficient to explain the emergence of *P. falciparum* as a human pathogen. Accumulating evidence suggests that RH5, EBL165, other members of the RH5 complex, invasion proteins, and possibly exported proteins that remodel the infected erythrocyte surface all contribute to host specificity and tropism. Gene transfers between *Plasmodium* species have played an important role in the parasite evolution and host-parasite interactions (Plenderleith et al., 2019).

## Host-Parasite Interaction and Evolution

### Highly Polymorphic Immune Targets

**Figure 3:**
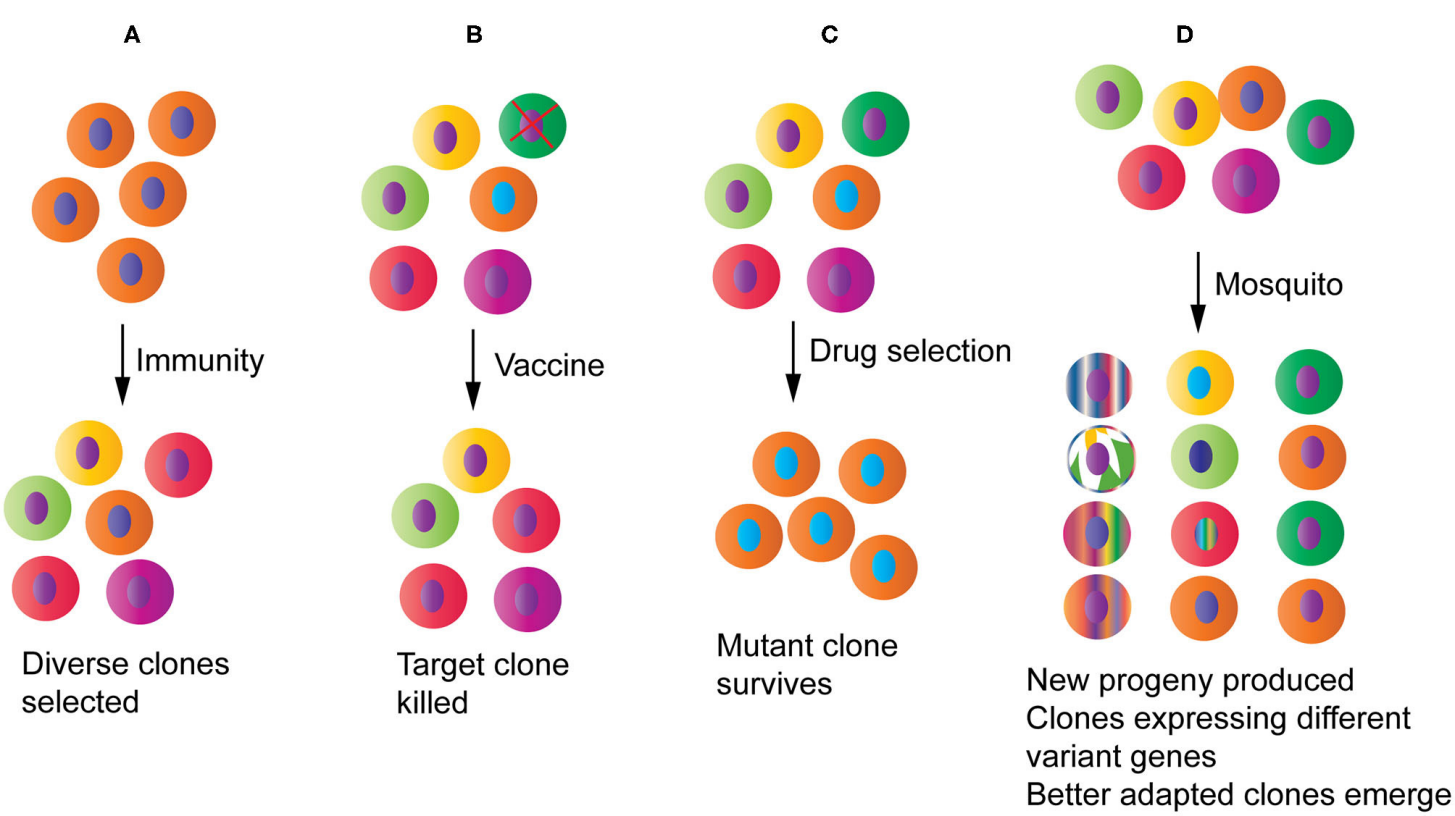
Potential impacts of host-parasite interactions and human intervention measures on parasite populations and evolution. **(A)** Host immunity will pressure parasites to diversify and/or express different sets of variant antigen genes, increasing diversity at immune targets. **(B)** A partially protective vaccine may selectively remove a specific parasite strain from the population. **(C)** Drug treatment will reduce population diversity by removing the drug sensitive parasites and selecting for one or a few drug resistant parasite clones that may then spread to many endemic regions. **(D)** Parasites passing through mosquitoes may generate new progeny through genetic recombination and chromosomal reassortment, reset gene expression profile particularly those related to parasite survival, and select for clones that are better adapted to specific mosquito species.

Malaria parasites trigger an immune response the moment when they enter a host. To survive in this hostile environment, the parasite displays a range of strategies to evade host killing mechanisms, including variations in antigen epitopes targeted by host immune machinery and interference or suppression of specific arms of the host immune response (Recker et al., 2011; Yam and Preiser, 2017; Larsen et al., 2018; Xia et al., 2018). One of the consequences of these host-parasite interactions is increased genetic diversity at genes encoding proteins under host immune selection (Figure 3A), leading to genetic signatures of diversifying selection in the parasite genome. Some highly polymorphic genes include those encoding proteins such as the apical membrane antigen 1 (AMA1), merozoite surface protein 1 (MSP1), and circumsporozoite protein (CSP). These highly polymorphic genes as well as genome-wide polymorphisms such as single nucleotide polymorphisms (SNPs) and microsatellites (MSs) have been used for genotyping parasite strains, tracking parasite migration and disease outbreak, studying parasite molecular evolution, and evaluating host immune response and vaccine efficacy (Ranjit and Sharma, 1999; Volkman et al., 2001, 2007; Mu et al., 2002; Wootton et al., 2002; Joy et al., 2003; Jeffares et al., 2007; Ouattara et al., 2010; Drew et al., 2012; Tanabe et al., 2015; Dewasurendra et al., 2018; Stone et al., 2018). The high levels of genetic polymorphisms in immune targeted genes also provide signatures to identify potential vaccine candidates, particularly signatures of balancing selection within parasite populations (Mu et al., 2007; Tetteh et al., 2009; Ochola et al., 2010; Weedall and Conway, 2010; Amambua-Ngwa et al., 2012; Conway, 2015). For example, Tetteh et al. (2009) compared methods for prospectively identifying genes under balancing selection using 26 genes known or predicted to encode merozoite surface-exposed proteins and concluded that significant evidence of balancing selection could be detected using Hudson-Kreitman-Aguade (HKA) and Tajima's D (TjD) tests. In another study, high throughput sequencing of 65 clinical isolates from an endemic Gambian population identified 337 genes with at least three SNPs that had Tajima's D values > 0, including 25 genes with Tajima's D values > 1.5 (Amambua-Ngwa et al., 2012). However, the use of highly polymorphic antigens as vaccines for protection against malaria is challenging because antibody responses to these molecules are generally allele-specific or strain-specific (Graves et al., 2016; Laurens et al., 2017), which represents one of the major roadblocks for developing an effective malaria vaccine.

### Diversity and Evolution of Gene Families of Malaria Parasites

#### The *var* Genes

In addition to highly polymorphic antigen genes, there are many polymorphic gene families in the *Plasmodium* genomes such as the *P. falciparum var* genes, SICA (Schizont Infected Cell Agglutination) *var* (SICAvar) genes in *P. knowlesi*, and the *Plasmodium* interspersed repeat (*pir*) multigene family, which include *P. falciparum* repetitive interspersed (*rif*) and subtelomeric variant open reading frame (*stevor*), found in all *Plasmodium* species (Su et al., 1995; Cunningham et al., 2010; Jemmely et al., 2010; Wahlgren et al., 2017; Galinski et al., 2018). Indeed, large multigene families are present in many *Apicomplexa* parasites (Reid, 2015). These gene families evolve at high rates and play critical roles in antigenic variation and immune evasion. The *var* genes encode *P. falciparum* erythrocyte membrane protein 1 (PfEMP1) proteins (Howard et al., 1988; Baruch et al., 1995; Su et al., 1995). Switches in expression among an estimated 60 *var* genes in every single parasite have been shown to correlate with variation in antigenic determinants that bind to different host receptors such as ICAM-1, CD36, EPCR, and other receptors (Smith et al., 1995; Wahlgren et al., 2017; Jensen et al., 2020). The binding of PfEMP1 proteins to host receptors is responsible for cytoadherence of infected erythrocytes (iRBCs) and the pathogenicity of severe malaria, particularly cerebral malaria (Smith et al., 2013; Jensen et al., 2020). The *P. falciparum* parasite evades host immunity via mutually exclusive expression (e.g., only one PfEMP1 is expressed on the surface of an iRBC at a time) of the highly diverse *var* family representing an almost unlimited gene pool at the parasite population level (Su et al., 1995; Chen et al., 1998; Hviid and Jensen, 2015; Deitsch and Dzikowski, 2017). Switches of *var* gene expression have been demonstrated in infections of human volunteers (Peters et al., 2002; Bachmann et al., 2019). Several mechanisms to explain the mutually exclusive *var* expression have been reported, including: (1) cis-acting DNA elements (promoters, silencers, activators) and anti-sense RNA transcripts acting to silence or activate the *var* genes; (2) epigenetic regulation through the presence or absence of specific epigenetic marks; (3) locations of the *var* genes in specific subnuclear compartments; (4) coordination between members of the *var* family to ensure activation of a new gene while simultaneously silencing of the previously active gene at the same time (Deitsch and Dzikowski, 2017). Similarly, there are also several potential mechanisms for the generation of new *var* genes and maintenance of the *var* gene repertoire. Although each parasite carries a set of approximately 60 *var* genes, a parasite strain usually has a unique set of *var* genes with diverse sequences that differ from those of other strains (Trimnell et al., 2006; Chen et al., 2011; Claessens et al., 2014; Day et al., 2017; Ruybal-Pesantez et al., 2017). In fact, it was reported that every child infected with *P. falciparum* had a unique *var* DBLα repertoire (Ruybal-Pesantez et al., 2017), and some specific types of homologous blocks or motifs have been associated with severe diseases (Avril et al., 2013; Rorick et al., 2013). Considering frequent mixed infection of multiple parasite strains in an individual patient in endemic regions (Conway et al., 1991; O'Brien et al., 2016; Zhu et al., 2019), the *var* gene repertoire in the parasite populations of an individual patient or in a village can be quite large.

What are the forces or mechanisms driving the high diversity of the *var* genes? Since the PfEMP1 proteins are expressed on the iRBC surface, they are expected to be under strong immune selection. In addition to switches in expression to different *var* genes, one obvious mechanism to evade immune recognition is substitutions of critical amino acids at antigenic epitopes. A second mechanism for generating *var* gene diversity is the production of hybrid copies of *var* genes through genetic recombination during meiosis in the mosquito host. If genetic recombination is the major driving force, then sequencing parasite strains in mixed infections from an individual patient would reveal shared haplotypes and breakpoints in some *var* genes among parasite strains from a mixed infection. Interestingly, transmission through a mosquito also resets the *var* gene switching process (Peters et al., 2002), suggesting that going through mosquitoes or a sexual cycle can change *var* gene expression profile and may also alter *var* gene composition in the genome at the same time. A third mechanism is generation of recombinant *var* genes through somatic recombination during asexual replication. Recombination events such as duplicative transposition of specific sequences were observed during parasite transformation in *in vitro* culture (Frank et al., 2008). By constructing large parasite clone trees and performing whole genome sequence analysis of *var* gene sequences in asexually replicating parasites, Claessens et al. (2014) found that the *Var* exon 1 recombined in a rate up to 0.2% of infected erythrocytes *in vitro* per life cycle, suggesting that *var* gene sequence polymorphism could be mainly generated during the asexual part of the life cycle (Claessens et al., 2014). Using targeted DNA double-strand breaks (DSBs) and long-read whole-genome sequencing, Zhang et al. (2019) showed that a cascade of recombination events could occur starting at a single DNA break within a subtelomeric region generating multiple new *var* genes (Zhang et al., 2019).

What is the mechanism mediating somatic recombination? One possible way of generating recombinant *var* genes during the asexual cycle is through microhomology-mediated end joining (MMEJ), involving alignment of microhomologous sequences flanking a DNA break and subsequent removal of the intermediate sequences (Grajcarek et al., 2019). Because of the absence of enzymes necessary for non-homologous end joining in *Plasmodium*, repair of DNA breaks generally required homologous templates (Lee et al., 2014). However, an end joining pathway for repair of DSBs without the presence of homologous sequence was observed in *P. falciparum*, with the repair junctions frequently containing short insertions in the surrounding sequences (Kirkman et al., 2014), suggesting that MMEJ may play a role in these events. Various enzymes for MMEJ are indeed present in *P. falciparum* parasites (Lee et al., 2014). Recently, MMEJ was shown to function in the *P. yoelli* genome and was able to generate a large number of deletion or insertion mutants of varying sizes in the central repeat region of *P. yoelii* CSP gene (Xu et al., 2019). Therefore, homologous regions among the different copies of the *var* genes may act as templates during DSB repair, leading to deletion, insertion, and translocation of repetitive DNA sequences and rapid evolution of new *var* genes.

The *var* genes have a GC content much higher than those of other protein coding genes in the *P. falciparum* genome (~34% vs. 20%), and how the genes with high GC content originated and are maintained in a genome with a high AT content remains largely unknown (Su et al., 1995; Gardner et al., 2002). One potential explanation is that the *var* gene came from the parasite hosts (humans and mosquitoes) through horizontal gene transfer; however, there is no evidence that the host genomes contain genes with structures and/or protein motifs observed in the PfEMP1. The *var* genes are not even present in the other human malaria parasites such as *Plasmodium vivax*, whose genome has high GC content in its genome (Carlton et al., 2008; Chan et al., 2015). Because *var* genes contain Duffy binding-like (DBL) domains that are also present in another gene family encoding erythrocyte invasion ligands (EBL proteins), it has been proposed that the *var* genes might have originated from the EBL genes (Smith et al., 2013). The *var* genes are also present in the *Laverania* parasites infecting African great apes (*P. praefalciparum, P. blacklocki, P. adleri, P. billcollinsi, P. gaboni*, and *P. reichenowi*) (Rask et al., 2010; Otto et al., 2014b, 2018; Laurens et al., 2017). Therefore, the *var* genes were already present when *P. falciparum* diverged from *P. preafalciaprum* to emerge as a human parasite 40,000–60,000 years ago (Otto et al., 2018). The question as how the *var* genes initiated in the ancestor remains unanswered. If *var* genes were derived from the genes containing DBL domains, why similar gene family expansion events did not occur in other malaria parasite species?

#### The *pir* Genes

Similar to the *var* genes, the *pir* multigene family is likely under host immune pressure and plays an important role in immune evasion (Cunningham et al., 2010). The *pir* gene family derives its name from the first initial of the parasite species name followed by “ir,” an abbreviation for interspersed repeat: *yir* for *P. yoelii* genes; *bir* for *P. berghei* genes; *vir* for *P. vivax* genes, and so on (Cunningham et al., 2010). Gene families of *stevor, rif*, and PfMC-2TM in *P. falciparum* were classified as *pir* gene families because of similar gene structures and sequence similarity in some introns (Janssen et al., 2002). The *pir* genes have a conserved three-exon structure, including a short first exon, a long second exon encoding conserved cysteine residues and a transmembrane domain, and a conserved third exon encoding cytoplasmic domain (Janssen et al., 2002). The *pir* genes are mostly distributed in the subtelomeric regions of chromosomes (except *kir* in *P. knowlesi*) with gene copies numbering from a few dozen to more than 800 (Cunningham et al., 2010). Distinct from the *var* genes, up to 40% of the *cir* gene repertoire are expressed throughout the intraerythrocytic cycle of development of the *P. chaboudi* parasite during infection (Lawton et al., 2012). The timing and level of transcription differs between *cir* genes, with ring, trophozoites, and schizonts expressing different sets of major *cir* genes (Cunningham et al., 2009). Additionally, individual CIR proteins were observed within differential localizations of iRBCs, including on the surface of iRBCs and merozoites, some of which could bind mouse erythrocytes and play a role in immune evasion (Yam et al., 2016; Yam and Preiser, 2017).

The mechanisms for generating *pir* gene diversity are expected to be similar to those of *var* genes, including amino acid substitutions at specific antigen epitopes in response to immune pressure and the generation of new recombinant genes through genetic recombination as well as MMEJ mediated somatic deletion, insertion and translocation. Genes with mosaic sequences or two genes with segments of 100% identity in the *P. yoelii* genome have been noted, suggesting crossovers between two copies of *pir* genes (Cunningham et al., 2010). However, rodent malaria parasites may have a lower degree of antigen diversity and undergo significantly less ectopic recombination than *P. falciparum*, partly due to the lack of components of the translesion (TLS) polymerases that are required for the recombination events that drive diversification of the multicopy gene families (Kirkman and Deitsch, 2020; Siao et al., 2020). Further discussions on functions of the *pir* genes can be found here (Cunningham et al., 2010; Su et al., 2019). More studies are necessary to better understand the mechanisms of gene expression regulation and the maintenance of genetic diversity of the *pir* genes.

### The EBL Genes and Evolution of Virulence in Laboratories

One of the consequences of host immune pressure on parasites is the generation of parasites with altered virulence. Whereas it has been difficult to evaluate how host immune response or vaccination impacts parasite evolution and virulence in humans, changes in parasite growth and virulence can be measured in rodent malaria parasites in the laboratory. One example is the *P. yoelii* EBL protein (PyEBL) that is known to bind the Duffy antigen/receptor for chemokines (DARC), a transmembrane glycoprotein expressed on epithelial cells, endothelial cells, and erythrocytes (Swardson-Olver et al., 2002; Woolley et al., 2005; Culleton and Kaneko, 2010). Whereas several genes encoding proteins with DBL domains have been found in *P. falciparum* such as PfEBL-140, PfEBL-175, and PfEBL-181, there is only a single copy of the EBL gene in *P. yoelii* (Culleton and Kaneko, 2010). PyEBL contains a single DBL domain, unlike other EBLs which may have two DBL domains per molecule.

Immunity or host response to malaria infection is generally parasite strain specific (Fluck et al., 2004; Cheesman et al., 2006; Early et al., 2018). The principle of strain specific immunity has been applied to identify immune targets in rodent malaria parasites using a strategy called linkage group selection (LGS) (Culleton et al., 2005, 2011; Pattaradilokrat et al., 2009; Cheesman et al., 2010; Abkallo et al., 2017). In a LGS analysis, phenotype-specific selection pressure such as immunity is applied to uncloned progeny of a genetic cross between two malaria parasites that differ in phenotype (strain specific immunity in this case) (Culleton et al., 2005; Cheesman et al., 2010). PyEBL has also been identified by LGS experiments because it plays a role in parasite invasion of RBCs and is also the target of host immunity (Pattaradilokrat et al., 2009; Abkallo et al., 2017). Mutations in the PyEBL gene not only affect parasite growth and virulence, but also influence iRBC surface protein and host immune response (Otsuki et al., 2009; Peng et al., 2020). There are isogenic pairs of rodent malaria parasites that have different parasite growth rates in mice and produce very different diseases. *P. yoelii yoelii* 17X (or YM) infection of BALB/c or C57BL/6 mice causes host death within 7 days post infection (pi), whereas its isogenic strain *P. y. yoelii* 17XNL (or 17X) is cleared by the host around 21 days pi (Otsuki et al., 2009; Pattaradilokrat et al., 2009). Both parasites were derived from *P. yoelii* 17X during passages in separate laboratories (Pattaradilokrat et al., 2008). A replacement of Cys to Arg (at position 731 or C731R) at the second Cys position in region 6 of PyEBL was found to be the major determinant for the difference in virulence and parasite growth; however, this mutation cannot explain the entire parasite growth phenotype (Otsuki et al., 2009). A second example is the isogenic parasites of *P. y. nigeriensis* N67 (N67) and *P. y. nigeriensis* N67C (N67C) that also have very different disease phenotypes in C57BL/6 mice, including parasitemia, tissue pathology, and host mortality (Wu et al., 2014; Lacerda-Queiroz et al., 2017). Parasitemia in C57BL/6 infected with N67 (1 × 10^6^ iRBCs) increases to ~40–50% day 5 pi, declines to below 10% on day 7 pi, and increases to ~60% at day 15 pi (Wu et al., 2014). The N67 infected mice die at ~day 20 pi. Mice infected with N67C have ~50% parasitemia day 6 pi and begin to die day 7 pi with declining parasitemia (Lacerda-Queiroz et al., 2017). Similarly, a substitution of Cys to Tyr (C741Y) in the protein trafficking domain of PyEBL between N67 and N67C parasites was found to influence disease phenotypes and host mortality (Peng et al., 2020). The substitution alters processing and trafficking of the PyEBL protein, reduces PyEBL binding to Band 3 of the RBC, increases phosphatidylserine (PS) surface exposure on iRBCs, and enhances iRBC osmotic fragility. Interestingly, the two studies have different conclusions on the mechanism of how the PyEBL substitutions affecting disease phenotypes, although the C731R and C741Y substitutions both appear to change PyEBL protein trafficking, from microneme localization to dense granules in the cytoplasm. For C731R substitution between 17X and 17XL (YM), parasite virulence was mostly attributed to altered erythrocyte invasion (Otsuki et al., 2009). In contrast, in the C741Y substitution in PyEBL between N67 and N67C was found to modulate host recognition of iRBCs and immune responses through interaction with RBC membrane molecules such as Band 3 and PS (Peng et al., 2020). A third example is the isogenic parasites *P. berghei* ANKA and *P. berghei* NK65: *P. berghei* ANKA infection is lethal with cerebral malaria symptoms and has been used as an experimental cerebral malaria (ECM) model, whereas *P. berghei* NK65 infection generally does not have neurological symptoms (Lacerda-Queiroz et al., 2011; Nacer et al., 2014). Even cloned lines of *P. berghei* ANKA can differ in their ability to induce ECM in the same host (Amani et al., 1998). However, the molecular mechanisms responsible for the differences in disease phenotype and virulence among these parasites are not clear. Additionally, the substitutions in PyEBL are likely not the only genetic changes contributing to the total disease phenotypes. Unknown differences including SNPs and variation in genes such as the *pir* multigene gene family may also contribute the differences in disease phenotypes and virulence (Cunningham et al., 2010). These parasite pairs are examples of the evolution of parasite genomes and changes in virulence following laboratory passages. There are additional parasite species and strains, including *P. chabaudi* and *P. vinckei*, that showed changes in parasite genome and virulence (Perkins et al., 2007). It can be expected that these types of phenotypic changes in parasite growth, disease virulence, and drug response are occurring daily in the huge parasite populations in endemic regions of the world. Additionally, mixed infections of multiple *P. falciparum* strains may lead to higher proportion of severe anemia, pulmonary complications, and multiple organ failure than those with single-strain *P. falciparum* infection (Kotepui et al., 2020). The dynamics of parasite genetic variation and gene expression are major roadblocks for developing effective vaccines and therapies.

## Parasite Selection on the Host Genome

Whereas host immunity is one of the major factors driving parasite evolution, malaria parasites and many other pathogens also have tremendous impacts on the adaption and evolution of their hosts (Daub et al., 2013). The well-known Haldane malaria hypothesis suggests an evolutionary advantage of the thalassemic condition in protection against malaria (Akide-Ndunge et al., 2003; Canali, 2008). Many proteins expressed on the RBC surface such as sickle cell HbS variants, DARC, and glycophorin A (GYPA), and proteins that play a role in host immune response such as TNF-α and IFN-γ have been associated with protection from severe complications of malaria (Carter and Mendis, 2002; Kwiatkowski, 2005; Verra et al., 2009; Hedrick, 2012; Kariuki and Williams, 2020). The protective genes were mostly identified through association studies including genome-wide association studies (GWAS). For example, a recent GWAS study performed in a Tanzanian population revealed association of protective roles of sickle cell HbS variant, interleukin receptors (IL-23R and IL-12RBR2), and the kelch-like protein KLHL3 (Ravenhall et al., 2018). In another study, Ebel et al. analyzed mammalian adaptation in 490 *Plasmodium*- or Piroplasm-interacting proteins (PPIPs) and showed that blood parasites imposed specific selection on PPIPs, leading to a higher number of adaptive substitutions in the PPIPs than expected throughout mammalian evolution (Ebel et al., 2017). Interestingly, many immune PPIPs were also enriched for viral and bacterial interactions, with 48% of the PPIPs interacting with viruses and/or bacteria. Moreover, there was a 2.5X enrichment of adaptation when only PPIPs interacting with *Plasmodium* or Piroplasms were considered. Malaria parasites interact with a large number of host genes (Wu et al., 2015, 2020), which may enhance adaptive substitutions in these genes. There are many excellent reviews on the effects of parasite infections, particularly malaria parasite infections, on host genome evolution (Tishkoff and Williams, 2002; Kwiatkowski, 2005; Mangano and Modiano, 2014; Goheen et al., 2017).

## Impact of Vaccines, Drugs, and Vectors on Parasite Populations and Disease

### Types of Malaria Vaccines

Developing an effective vaccine has been one of the major goals of malaria research for more than 40 years, and we are still far away from achieving the goal. Malaria vaccines can be divided into several categories based on the parasite developmental stages such as pre-erythrocytic vaccines, blood stage vaccines including those against pregnancy-associated malaria, transmission-blocking vaccines, multistage or multiantigen vaccines, and whole organism based vaccines (Frimpong et al., 2018; Laurens, 2018; Beeson et al., 2019). Development of a cross-species vaccine that protects against multiple *Plasmodium* species or strains is an important goal. *P. falciparum* 3D7 strain has been the model strain for studies of genome structure and analyses of transcriptome and proteome. Subsequently, the majority of vaccines are based on sequences from the 3D7 parasite; however, the 3D7 strain is often not the most prevalent strain in malaria-endemic areas. Currently, the leading vaccine candidates are mostly based on polymorphic antigens with the vaccine target representing only a single variant strain. However, immunity to malaria is generally strain-specific, and the development of an effective vaccine is often hampered by strain-specific and/or partial immunity generated by vaccination.

### Partially Protective Vaccines and Effects on Host Populations

The most promising and extensively tested malaria vaccine candidate against *P. falciparum* parasites is RTS,S/AS01 that consists of 18 copies of the central repeat and the C-terminal domain of PfCSP fused to hepatitis B virus surface antigen (HBsAg) (Draper et al., 2018). In a large-scale phase III trial involving 15,460 children, including 6,537 infants aged 6–12 weeks and 8,923 children aged 5–17 months, vaccine efficacy (VE) against clinical malaria was reported to be ~50–56%, and VE against severe malaria in the combined age children was ~35% in the per-protocol population at 11 month follow-up (Rts et al., 2011). However, in a 7 year follow-up analysis, the VEs of RTS,S/AS01 were estimated to be 35.9% in the first year after vaccination, 2.5% in the fourth year, and a negative VE in the fifth year (Olotu et al., 2016). More generalized convulsive seizures were observed in the RTS,S vaccinated group than the control group (comparator rabies vaccine group) in the older children (5–17 months) within 7 days after RTS,S/AS01 vaccination (Rts et al., 2011). Additionally, significantly more cases of meningitis were reported in the older children (Rts et al., 2012; Rts, 2015). Moreover, RTS,S vaccine was later found to be associated with significantly higher all-cause mortality in girls of both age groups (Klein et al., 2016). The higher rates of meningitis and mortality in girls have prompted the World Health Organization (WHO) to issue a position paper cautioning on malaria vaccines, with emphasis on the RTS,S/AS01 (World Health Organization, 2018) and discussions on the need of further studies on vaccine safety and negative non-specific effects (Aaby et al., 2015; Muller et al., 2015). Higher all-cause mortality was also observed in girls after vaccination with the high-titer measle vaccine (HTMV) (Aaby et al., 1993), which was later found to be likely associated with diphtheria-tetanus-pertussis (DTP) vaccine or interaction between HTMV and DTP (Aaby et al., 2003, 2007). Generally, women have a stronger cellular and humoral responses to infections and are more likely to have autoimmune diseases due to X-linked genes, hormones, and societal context (Fish, 2008; Billi et al., 2019). The observation of higher rate of severe symptoms in RTS,S vaccinated girls could be due to enhanced immune reactions from vaccine priming of the immune system.

A partial protective vaccine with enhanced adverse effects brings out some important issues in malaria vaccine development that has largely been ignored so far: Can a partially protective malaria vaccine prime for more severe disease symptoms and select for parasite population “resistant” to specific vaccines? The T-cell prime hypothesis proposed to explain clinical malaria and antibody dependent enhancement (ADE) observed in many viral infections may provide some answers to the questions (Porterfield, 1986; Riley, 1999). More than 20 years ago, Dr. Eleanor Riley proposed that T-cell priming is required for amplification of the inflammatory response to malaria, which can explain the patterns of clinical malaria in both endemic and non-endemic populations (Riley, 1999). According to the hypothesis, primary infections would induce low levels of IFN-γ and TNF-α; at the same time, antigen-specific T cells are primed by the infection. On reinfection, large amounts of cytokines such as IFN-γ and TNF-α are produced by the primed T cells, leading to an increased risk of cerebral malaria or other severe symptoms. She also cautioned that vaccinations with BCG or tetanus toxoid might prime T cells to respond to malaria antigens and suggested that having an appropriate balance between protective and pathogenic levels of Th1-derived cytokines will be crucial for a successful outcome of a vaccine (Riley, 1999). A partial protective vaccine may also prime the immune system in some individuals that can mount a strong inflammatory response when infected, leading to enhanced severe symptoms. Another related possibility is ADE that was observed in various viral infections, including Dengue virus (Waggoner et al., 2020), influenza (Winarski et al., 2019), Zika virus (Asad et al., 2019), coronavirus (Negro, 2020; Wan et al., 2020), and *Acinetobacter baumannii* bacteria (Wang-Lin et al., 2019). ADE is a well-known mechanism that viruses or bacteria may infect susceptible cells by interacting with antibodies or complement components (Fc or complement receptors), leading to enhanced viral entry into host cells and/or higher replication rate. Cross-reactive IgGs can opsonize viral particles by binding the virus through Fc receptors on host cell surface and may also mediate immune suppression to further increase viral load (Halstead et al., 2010). Monoclonal antibodies have been shown to destabilize the influenza HA stem domain, leading to faster kinetics of influenza virus fusion and enhanced respiratory disease (Winarski et al., 2019). Antibody enhancement of malaria transmission and complement activation in conjunction with antibodies in enhancing RBC invasion have been reported too (Biryukov et al., 2016; Stone et al., 2019). The RTS,S vaccine might prime T cells in some hosts and/or produce antibodies that can amplify immune response and enhance clinical symptoms. Addition of an arm to evaluate the potential of a vaccine to enhance adverse effects should be included before or during a vaccine trial.

### Effects of Vaccination on Parasite Population and Gene Expression

Malaria parasite populations in endemic regions consist of a large number of strains that express different alleles of vaccine target proteins. Due to technical limitation, malaria vaccines are usually designed based on sequences from one to a limited number of alleles of a vaccine target. It is has been shown that anti-malaria immunity is mostly strain-specific (Cheesman et al., 2006; Graves et al., 2016; Laurens et al., 2017; Early et al., 2018), and a vaccine based on a limited number of alleles of a target may select for parasite populations with alternative alleles. Indeed, vaccination with pneumococcal conjugate vaccines (PCVs) changed the pneumococcal populations in children, resulting in statistically significant shift from vaccine-type population to non-vaccine-type populations (Quirk et al., 2018). Similarly, using the rodent malaria model *P. chabaudi* and recombinant AMA-1 antigen, it was shown that mono-allelic immunization increased the frequency of heterologous clones in mixed clone infections (Barclay et al., 2008). Moreover, analysis of parasite genotypes collected from the RTS,S phase III trial showed that the RTS,S vaccine had greater activity against malaria parasites with the matched PfCSP allele than against those with mismatched alleles (Neafsey et al., 2015). Therefore, vaccination with an allele of the target antigen will likely change target allele proportion in parasite populations, and large-scale vaccination may lead to vaccine mediated depletion of specific alleles targeted by the vaccine (Figure 3B). The impacts of this type of selection on parasite populations, including the possibility of selecting more virulent parasite strains, remain largely unknown and required further investigations. Interestingly, a vaccine may also alter parasite expression of variant antigen genes. Vaccination of 10 African volunteers with the PfSPZ vaccine showed parasites from individuals with intermediate antibody levels expressed only few *var* gene variants, whereas those with low antibody levels expressed a broad spectrum of multiple, predominantly subtelomeric *var* genes coding PfEMP1 binding to endothelial protein C receptor (EPCR) that is associated with severe childhood malaria (Bachmann et al., 2019). Therefore, vaccination certainly can impact parasite population dynamics, genetic diversity, and possibly virulence.

### Drug Selection on Parasite Populations and Evolution

In addition to host immune response, antimalarial drugs have played a significant role in shaping parasite populations. Application of antimalarial drugs puts pressure on parasite populations and likely will select parasites with mutations that can confer resistance to the drugs, leading drug selective sweeps (Figure 3C). Some examples of drug selective sweep include mutations in *P. falciparum* chloroquine resistance transporter (PfCRT) and in dihydrofolate reductase-thymidylate synthase and dihydropteroate synthase (PfDHFR-TS and PfDHPS) conferring resistances to chloroquine and pyrimethamine/sulfadoxine (PS), respectively (Peterson et al., 1990; Fidock et al., 2000). Under drug pressure, a small number of parasites with resistant mutations will survive, whereas parasites without the mutations are killed by the drugs. With large-scale drug use, parasites with resistant mutations will spread, replacing parasites sensitive to the drugs. Indeed, worldwide chloroquine and PS selective sweeps have been reported (Wootton et al., 2002; Roper et al., 2004; McCollum et al., 2008), which can greatly reduce population diversity in many endemic areas generating population bottlenecks. Drug selective sweeps are important factors in shaping the current worldwide parasite populations and may have contributed to Malaria's Eve hypothesis debate (Rich et al., 1998; Su et al., 2003). The sweeps of parasites derived from one or a few mutants conferring resistance to a drug will result in a relatively homogeneous parasite population (Figure 3C), as was seen in the Brazilian Amazon after wide spread use of chloroquine (Wootton et al., 2002). A relatively homogeneous parasite population may be advantageous for vaccine development.

### The Role of Mosquito Vectors in Altering Parasite Populations

Mosquito vectors can shape parasite populations in many ways (Figure 3D). First, genetic changes such as chromosome reassortment and crossover will occur inside a mosquito and generate new parasite strains if a patient is infected with two or more variant strains, which will increase parasite population diversity. A high multiplicity of infection is frequently observed in patients in regions with high transmission intensity (Kobbe et al., 2006; Felger et al., 2012). Second, parasite transmission through mosquitoes can also re-program gene expression profiles. For example, changes in composition and frequency of *var* gene transcripts were observed between cultured *P. falciparum* parasites used to infect mosquitoes and the parasites recovered from infected volunteers after mosquito bites, suggesting re-programing *var* gene expression profile (Peters et al., 2002). Similarly, attenuated parasite virulence was found to be associated with modified expression of the *pir* multi-gene family. Transmission of *P. c. chabaudi* through the mosquito changes gene expression of the *pir* multi-gene family in the erythrocytic cycle, leading to changes in parasite virulence and immune response in the mammalian hosts (Spence et al., 2013). Third, vector competency can also influence parasite transmission frequency. A study of *P. vivax* populations in southern Mexico showed that the distributions of parasite populations largely mirrored those of mosquito species, which was confirmed by feeding experiments in the laboratory (Joy et al., 2008). The results suggest a reciprocal selection between malaria parasites and mosquito vectors and local adaptation of the parasites to mosquito species in different environments (coastal or hill sites). *P. falciparum* isolates from Africa, Asia, or the Americas were shown to have low compatibility to malaria vectors from different continents, which is mainly mediated by a parasite protein called Pfs47 due to its interaction with mosquito immune system (Molina-Cruz et al., 2015). Mosquito phagocytes play an important role in mosquito vector competence and immunity against malaria infection (Kwon and Smith, 2019). Additionally, *Serratia marcescens* bacteria isolated from either laboratory-reared mosquitoes or wild populations in Burkina Faso shows great phenotypic variation that is directly correlated with its ability to inhibit *Plasmodium* development within the mosquito midgut (Bando et al., 2013).

## Approaches for Studying Host-Parasite Interactions

### Identification of Host-Parasite Interacting Genes Using Genetic Crosses

In addition to antibody response to antigens expressed on the surfaces of iRBCs, malaria infections also stimulate differential activation of immune cells and the production of cytokines and chemokines as part of the innate immune response. What are the parasite ligands that initially trigger host innate responses? What are the host receptors that recognize the parasite molecules? How are the host responses to malaria infections regulated? What are the impacts of the host-parasite interaction on the evolution of both the host and parasite genomes? As discussed above, host immunity drives diversity of parasite antigen genes, and parasite infections select for protective alleles in host response genes. Large numbers of parasite antigens and host protective genes have been identified (Kwiatkowski, 2005; Salinas et al., 2019; Vijayan and Chitnis, 2019; de Jong et al., 2020). However, the numbers of host and parasite molecules involved in host-parasite interactions are likely much larger than the numbers of genes so far investigated. For example, Wu et al. (2015) performed an immunogenetic screen using trans-species expression quantitative trait locus (ts-eQTL) analysis and significantly (LOD score ≥ 3.0) linked 1,054 host genes to a large number of parasite genetic markers/loci (Wu et al., 2015). In this study, microsatellite genotypes of the *P. yoelii* malaria parasite and phenotypes of host (mouse) gene expression levels day 4 pi were analyzed for significant linkages between parasite genetic markers and host gene expression levels. Additionally, host genes that were linked (LOD score ≥ 3.0 or 2.0) to parasite genetic loci were clustered based on genome-wide patterns of LOD scores (GPLSs), showing that genes functioning in related pathways generally have similar GPLSs. This study demonstrates that a large number of host genes respond to *P. yoelii* parasites as early as day 4 pi. Similarly, many parasite genetic loci were predicted to interact with host genes belonging to specific pathways. For example, a genetic locus on parasite chromosome 8 was significantly linked to a large number of host genes play a role in hematopoiesis, suggesting that a gene or genes in the locus may influence host hematopoiesis. The study represents the first QTL analysis involving two organisms, microsatellite genotypes from malaria parasites and gene expression as phenotypes from the host, with the advantage of identifying interacting genes from both the parasite and the host. This ts-QTL analysis is a novel approach for identifying interacting genes from both parasite and the host at the same time, taking advantage of genetic recombination and chromosome reassortment during the sexual stage of a pathogen in generating progeny with unique genomes inherited from the parents. In a follow-up study using mRNA isolated from the spleens of infected mice 24 h pi (earlier than day 4), many host receptors including Toll-like receptors (TLR), retinoic acid-inducible gene-I-like receptors (RLRs), G-protein coupled receptors (GPCRs), and olfactory receptors (ORs) were significantly linked to parasite genetic loci, providing important candidate genes for studying host molecules that recognize parasite ligands or pathogen-associated molecular patterns (PAMPs) (Wu et al., 2020). Ts-QTL holds the potential to identify more interacting genes using additional genetic crosses or host RNA samples collected at different time points throughout the parasite life cycle.

### Transcriptome Analysis of Patient Samples

Another approach to identify host gene responses to malaria infections is to use samples collected from malaria patients, although a study using patient blood samples can be influenced by many factors such as variations in infection time, host genetic background and immune status, mixed parasite infections, nutrition status, antimalarial drug usages, etc. Mixed strain infections can change parasite growth dynamics, relapse time (for *P. vivax*), disease severity, and host mortality rate (Lover and Coker, 2015; Kotepui et al., 2020). Similarly, the genetic composition of each patient is unique, and variations in host genetic background will also affect host responses. Therefore, dissecting host-parasite genetic interactions using patient samples has proven to be much more complicated than using infections in inbred mice with cloned progeny from a genetic cross.

As discussed above, GWAS has been successful in identifying some host genes that contribute to protection against malaria. As an alternative, analysis of host gene expression in response to malaria infection can be useful too, particularly when transcripts from both the host and parasites are analyzed at the same time using RNAseq (Greenwood et al., 2016). Using blood samples of *P. falciparum*-infected West African children, Idaghdour et al. (2012) performed a joint genome-wide analysis of gene expression and genetic polymorphisms, and showed that malaria infection and host genotype both influenced immune gene expression throughout the genome (Idaghdour et al., 2012). The study implicated many genes in the complement system, antigen processing and presentation, and T-cell activation in response to malaria infections (Idaghdour et al., 2012). For example, the authors noted that the expression of five genetically regulated human leukocyte antigen (HLA) class II loci was negatively correlated with parasite load and molecules such as IL18R1, TLR4, TLR5, IFNGR1, and IFNGR2, suggesting impairment of antigen processing and immune signaling. Impairment of antigen processing and T cell activation, including suppression of various MHCII genes, were also observed in C57BL/6 mice infected with different *P. yoelii* strains and progeny of a *P. yoelii* genetic cross (Xia et al., 2018). In a dual transcriptome analyses of the host and parasite genes in samples from 46 malaria-infected Gambian children, Lee et al. (2018) performed multivariate analyses and showed association of disease severity with increased expression of granulopoiesis and IFN-γ related genes as well as inadequate suppression of IFN-I signaling (Lee et al., 2018). In another analysis by the same group, lower expression of *cxcl10*, HLA genes, and IFN-I associated genes, and higher expression of cathepsin G and matrix metallopeptidase 9 genes were associated with parasite growth inhibition (Georgiadou et al., 2019). Moreover, a blood transcriptional analysis in Senegalese patients with cerebral malaria (CM), severe non-cerebral malaria (NCM), or mild malaria (MM) identified host gene clusters that discriminated between CM and MM patients, showing that genes involved in BCR-, TCR-, TLR-, cytokine-, FcεRI-, and FCGR- signaling pathways played a role in cerebral malaria (Thiam et al., 2019). Associations between disease severity and PfEMP1 transcripts and specific domains were also observed in RNAseq data from 44 parasite isolates that caused SM and MM malaria in Papuan patients (Tonkin-Hill et al., 2018). These studies illustrate that there are signatures of host transcriptional responses to malaria infections and disease severity. Indeed, blood transcriptome analyses of pathogen-specific host immune responses have been proposed as a tool for improving the diagnosis and classification of patients with infectious diseases (Mejias et al., 2014). However, among the studies of host transcriptional response to malaria infections in animal models and in humans, the results are not always consistent, likely due to differences in study design, study objective, transmission intensity, sample collection time, as well as lack of replication. Caution is warranted when interpreting the results from field-based analyses.

There are other approaches for studying host-parasite interaction such as analysis of genome diversity and genetic association (Early et al., 2018; Damena et al., 2019; Malaria Genomic Epidemiology Network, 2019), computational prediction of host-parasite interactomes (Wuchty, 2011; Cuesta-Astroz et al., 2019), metabolite profiling to investigate transcriptomic changes during malaria infection (Tang et al., 2018), and systems biology/integrative approaches to study host immune responses (Mahanta et al., 2018). These are just few emerging and promising examples using -omics data to study host-parasite interactions.

## Some Key Questions and Challenges

There are several area that require special attention: (1) The new *Laverania* species are identified through nucleic acid sequence analyses. Morphological and biological characterization of the parasites will be critical for establishing these parasites as solid independent species. (2) The molecular mechanism of host specificity of the *Laverania* species is interesting and requires further study. (3) The origin of the *P. falciparum var* genes remains unknown. How did this gene family with a high GC-content originated and how is it maintained in a AT-rich genome? (4) Similar to the *var* gene family, the origins and functions of the *pir* gene families require additional studies. The *pir* genes likely play important roles in virulence and immune evasion. (5) More studies are necessary to elucidate the mechanisms that generate and maintain diversity of the *var* and *pir* genes in a parasite cell. (6) The impacts of antimalarial drugs and vaccines on the diversity and evolution of malaria parasite populations are important issues related to disease control and management. (7) The long-term impacts of antimalarial drugs and vaccines on host health and immune system also require more attentions. ADE is a real possibility for malaria vaccines. (8) Insect vectors play important roles in the evolution, diversity and transmission of malaria parasites. This is an area that has not received sufficient attention. (9) The parasite molecules that trigger host innate immune responses remain largely uncharacterized. DNA and RNA have been implicated in stimulating type I interferon responses, and strain-specific responses have been observed. However, the specific sequences that can bind to host receptors and the mechanisms of the strain-specific innate immune responses require additional studies.

## Conclusion

Host-parasite interactions are the main forces driving the evolution of both malaria parasites and their hosts. Genome diversity and gene function have co-evolved shaping the current genomes of malaria parasites and their hosts. Application of antimalarial drugs, deployment of vaccines, modulation of host immunity, and the development in mosquito vector control measures all have the potential to alter parasite populations including genome diversity and virulence. Proper monitoring of parasite populations in the context of drug, vaccine, and vector control programs is necessary to minimize unintended negative impacts on parasite population dynamics and disease severity.

## Author Contributions

X-zS and DAJ conceive ideas and writing. CZ wrote and created Figure 1. All authors contributed to the article and approved the submitted version.

## Conflict of Interest

The authors declare that the research was conducted in the absence of any commercial or financial relationships that could be construed as a potential conflict of interest.

## Glossary

*cyt b*: cytochrome oxidase b gene
ADE: antibody dependent enhancement
AMA1: apical membrane antigen 1
CM: cerebral malaria
CSP: circumsporozoite protein
DBL: Duffy binding-like
CMAH: cytidine monophospho-N-acetylneuraminic acid hydroxylase
*dhfr-ts*: dihydrofolate reductase-thymidylate synthase gene
DTP: diphtheria-tetanus-pertussis
DARC: Duffy antigen/receptor for chemokines
EPCR: endothelial protein C receptor
EBL: erythrocyte binding-like
ECM: experimental cerebral malaria
GPCR: G-protein coupled receptor
GWAS: genome-wide association studies
GPLS: genome-wide patterns of LOD score
GYPA: glycophorin A
HBsAg: hepatitis B virus surface antigen
HTMV: high-titer measle vaccine
HKA: Hudson-Kreitman-Aguade
HLA: human leukocyte antigen
iRBC: infected erythrocyte
*IntRh5*: introgressed Rh5 gene
LGS: linkage group selection
MSP1: merozoite surface protein 1
MSP2: merozoite surface protein 2
MMEJ: microhomology-mediated end joining
MS: microsatellite
MM: mild malaria
OR: olfactory receptor
PfCRT: *P. falciparum* chloroquine resistance transporter
PfDHFR-TS: *P. falciparum* dihydrofolate reductase-thymidylate synthase
PfDHPS: *P. falciparum* dihydropteroate synthase
PfEbl165: *P. falciparum* EBL165 gene
PfEMP1: *P. falciparum* erythrocyte membrane protein 1
N67: *Plasmodium y. nigeriensis* N67
N67C: *Plasmodium y. nigeriensis* N67C
PyEBL: *Plasmodium yoelii* erythrocyte binding-like protein
PAMPs: pathogen-associated molecular patterns
PS: phosphatidylserine
PPIPs: Piroplasm-interacting proteins
*pir*: *Plasmodium* interspersed repeat
PCV: pneumococcal conjugate vaccine
RBC: red blood cell
*rif*: repetitive interspersed
RH5: reticulocyte-binding-like homologous protein 5
RLR: retinoic acid-inducible gene-I-like receptor
SICA: schizont infected cell agglutination
NCM: severe non-cerebral malaria
SNP: single nucleotide polymorphism
*stevor*: subtelomeric variant open reading frame
SPR: surface plasmon resonance
TjD: Tajima's D
CyRPA: cysteine-rich protective antigen
TLR: Toll-like receptors
Ts-eQTL: trans-species expression quantitative trait locus
VE: vaccine efficacy
WHO: World Health Organization.
